# A novel risk score to predict 1-year functional outcome after intracerebral hemorrhage and comparison with existing scores

**DOI:** 10.1186/cc13130

**Published:** 2013-11-29

**Authors:** Ruijun Ji, Haipeng Shen, Yuesong Pan, Penglian Wang, Gaifen Liu, Yilong Wang, Hao Li, Xingquan Zhao, Yongjun Wang

**Affiliations:** 1 Tiantan Comprehensive Stroke Center, Beijing Tiantan Hospital, Capital Medical University, No. 6 Tiantanxili, Dongcheng District, Beijing 100050, China; 2 Department of Statistics and Operation Research, University of North Carolina, Chapel Hill, NC, USA

## Abstract

**Introduction:**

Spontaneous intracerebral hemorrhage (ICH) is one of leading causes of mortality and morbidity worldwide. Several predictive models have been developed for ICH; however, none of them have been consistently used in routine clinical practice or clinical research. In the study, we aimed to develop and validate a risk score for predicting 1-year functional outcome after ICH (ICH Functional Outcome Score, ICH-FOS). Furthermore, we compared discrimination of the ICH-FOS and 8 existing ICH scores with regard to 30-day, 3-month, 6-month, and 1-year functional outcome and mortality after ICH.

**Methods:**

The ICH-FOS was developed based on the China National Stroke Registry, in which eligible patients were randomly divided into derivation (60%) and validation (40%) cohorts. Poor functional outcome was defined as modified Rankin Scale score (mRS) ≥3 at 1 year after ICH. Multivariable logistic regression was performed to determine independent predictors, and β-coefficients were used to generate scoring system of the ICH-FOS. The area under the receiver operating characteristic curve (AUROC) and Hosmer-Lemeshow goodness-of-fit test were used to assess model discrimination and calibration.

**Results:**

The overall 1-year poor functional outcome (mRS ≥ 3) was 46.7% and 44.9% in the derivation (n = 1,953) and validation (n = 1,302) cohorts, respectively. A 16-point ICH-FOS was developed from the set of independent predictors of 1-year poor functional outcome after ICH including age (P < 0.001), admission National Institutes of Health Stroke Scale score (P < 0.001), Glasgow Coma Scale score (P < 0.001), blood glucose (P = 0.002), ICH location (P < 0.001), hematoma volume (P < 0.001), and intraventricular extension (P < 0.001). The ICH-FOS showed good discrimination (AUROC) in the derivation (0.836, 95% CI: 0.819-0.854) and validation (0.830, 95% CI: 0.808-0.852) cohorts. The ICH-FOS was well calibrated (Hosmer-Lemeshow test) in the derivation (P = 0.42) and validation (P = 0.39) cohort. When compared to 8 prior ICH scores, the ICH-FOS showed significantly better discrimination with regard to 1-year functional outcome and mortality after ICH (all P < 0.0001). Meanwhile, the ICH-FOS also demonstrated either comparable or significantly better discrimination for poor functional outcome and mortality at 30-day, 3-month, and 6-month after ICH.

**Conclusion:**

The ICH-FOS is a valid clinical grading scale for 1-year functional outcome after ICH. Further validation of the ICH-FOS in different populations is needed.

## Introduction

Spontaneous intracerebral hemorrhage (ICH) accounts for 10% to 15% of all strokes and is one of the leading causes of stroke related mortality and morbidity worldwide [1-4]. Despite advances in medical knowledge, treatment for ICH remains strictly supportive. Debate continues over the development of a standardized and widely accepted clinical grading scale and outcome prediction model for ICH [5].

Several predictive models have been developed for ICH [6-16]; however, none of them have been consistently used in routine clinical practice or clinical research [5]. Moreover, the existing ICH scores were mainly designed for short-term (in-hospital or 30 days after ICH) [6-8,10,13-16] or intermediate-term (three months after ICH) [9,11] outcome prediction. Studies have shown that a substantial proportion of ICH patients continue to improve throughout the first year after ICH [17]. Although a few risk scores were developed [8,12] or reevaluated [17] for predicting long-term (six months or one year after ICH) functional outcome after ICH, they were not sufficiently validated.

In the present study, we aimed to develop and validate a risk score (ICH Functional Outcome Score, ICH-FOS) for predicting poor functional outcome at one year after ICH. Furthermore, we compared discrimination of the ICH-FOS and existing ICH scores with regard to 30-day, 3-month, 6-month, and 1-year poor functional outcome and mortality after ICH.

## Materials and methods

### Study population

The derivation and validation cohort originated from the largest stroke registry in China, the China National Stroke Registry (CNSR), which was a nationwide, multicenter, and prospective registry of consecutive patients with acute cerebrovascular events [18]. Briefly, hospitals in China are classified into three Levels: I (community hospitals); II (hospitals that serve several communities); and III (central hospitals for a certain district or city). A Level III hospital is usually an academic center and more medically advanced than level I and II hospitals. In total, 242 potential sites including 114 grade III, 71 grade II and 57 grade I hospitals, from both urban and rural areas, were initially identified by soliciting applications. The CNSR steering committee evaluated the research capability and commitment to the registry of each hospital with a preliminary survey. Finally, a total of 132 hospitals including 100 Level IIIs and 32 Level IIs, which cover 27 provinces and 4 municipalities across China, were selected. Trained research coordinators at each institute reviewed medical records daily to identify, obtain consent and enroll consecutively eligible patients. To be eligible for the study, subjects had to meet the following criteria: (1) age 18 years or older; (2) hospitalized with a primary diagnosis of spontaneous ICH according to World Health Organization criteria [19] and with computed tomography (CT) confirmation; (3) direct admission to hospital from a physician’s clinic or emergency department; and (4) written informed consent from patients or their legal representatives. Patients were excluded if any of the criteria below were met: (1) pre-stroke dependence (modified Rankin Scale score ≥3); (2) patients who did not agreed to participate in follow-up; and (3) patients who were lost to one-year follow-up. Due to the fact that documentation of hematoma volume in the CNSR was not mandatory, for this study, we also excluded patients whose admission hematoma volume was not available. Eligible patients for the study were randomly divided into derivation (60%) and validation (40%) cohorts. The scientific use of data registered in the CNSR was approved by the central institutional review board at Beijing Tiantan Hospital and local ethical committees. For a complete list of CNSR investigators and ethical committees, see Additional file 1: Appendix A and B.

### Data collection and definition of variables

In the CNSR network, a standardized case report form was used for data collection. The relevant data were prospectively recorded. For this study, the following candidate variables were analyzed: (1) demographics (age and gender); (2) stroke risk factors: hypertension (history of hypertension or anti-hypertensive medication use), diabetes mellitus (history of diabetes mellitus or anti-diabetic medication use), dyslipidemia (history of dyslipidemia or lipid-lowering medication use), atrial fibrillation (history of atrial fibrillation or documentation of atrial fibrillation on admission), coronary heart disease, history of stroke/transient ischemic attack (TIA), current smoking and heavy alcohol consumption (≥2 standard alcohol beverages per day); (3) transportation mode to hospital (by emergency medical system or private transportation); (4) pre-admission medications: anticoagulation treatment with warfarin, antiplatelet treatment and statins use; (5) admission stroke severity based on the National Institutes of Health Stroke Scale score (NIHSS) and the Glasgow Coma Scale (GCS) score; (6) admission systolic and diastolic blood pressure (mmHg): (7) admission laboratory tests: hemoglobin, white blood cell count, platelet count, blood glucose, and creatinine; (8) neuroimaging variables: intracerebral hemorrhage volume was measured using the ABC/2 method [20]. Hematoma location was classified as supratentorial or infratentorial ICH. The presence or absence of intraventricular extension was documented on the initial head CT as well. All images were prospectively viewed by a trained neuroradiologist blinded to clinical data at different study centers; and (9) hospital academic status (academic or non-academic).

### Functional outcome assessment

The modified Rankin Scale (mRS) was used to assess functional outcome at one year after ICH. A central follow-up blinded to baseline variables was made by telephone interview by trained interviewers based on a standardized interview protocol. Poor functional outcome was defined as mRS ≥3 at one year after ICH. Since different clinical care contexts or clinical research studies may define poor outcome differently, we also examined discrimination of the ICH-FOS for one-year functional outcome when mRS ≥4 and ≥5 were used to define poor functional outcome.

### Statistical analysis

Model building was performed exclusively in the derivation cohort. In univariate analysis, Chi-square and Mann–Whitney tests were used as appropriate. Logistic regression was used to determine independent predictors for poor functional outcome at one year after ICH. Candidate variables were those with a biologically plausible link to poor functional outcome after ICH on the basis of prior publication or those associated with poor functional outcome (mRS ≥3) on univariate analysis (*P* ≤0.1). On multivariable analysis, a backward stepwise method was used to remove nonsignificant variables from the model. To test for collinearity between the covariates of the final multivariable model, the tolerance and variance inflation factor (VIF) of each covariate was calculated. The β-coefficients from the final model were used to generate the scoring system of the ICH-FOS, as in previous studies [21,22]. To derive an integer value, the β-coefficient was multiplied by 4 and was rounded to the closest integer. The resulting ICH-FOS was then validated by assessing model discrimination and calibration in the validation cohort [23]. Discrimination was assessed by calculating the area under the receiver operating characteristic curve (AUROC). Calibration was assessed by the Hosmer-Lemeshow goodness-of-fit test and plot of observed versus predicted risk according to 10 deciles of the predicted risk.

Furthermore, we compared discrimination of the ICH-FOS and prior ICH scores for ICH outcomes in the overall cohort. Because prior ICH scores were designed for predicting ICH outcomes at different time points, in this study, we compared discrimination of the ICH-FOS and prior ICH scores with regard to 30-day, 3-month, 6-month, and 1-year poor functional outcomes (mRS ≥3) and mortality after ICH. The primary criterion for selection of the model was whether all elements required for the model were available in our dataset and, finally, eight existing ICH scores met this criterion (original ICH score, modified ICH score, Essen ICH score, ICH grading scale (ICH-GS) score, FUNC score, modified ICH (MICH) score, secondary ICH (sICH) score, and Landseed ICH score). The AUROC and maximum Youden Index were used to evaluate model discrimination. Pairwise AUROCs were compared using Delong’s method [24] and sensitivity, specificity, positive predictive value (PPV) and negative predictive value (NPV) were calculated at each ICH score’s maximum Youden Index.

All tests were two-tailed and statistical significance was determined at α level of 0.05. Statistical analysis was performed using SAS 9.1 (SAS Institute, Cary, NC, USA), SPSS 17.0 (SPSS Inc., Chicago, IL, USA), and Medcalc software 12.3 (MedCalc®).

## Results

### Patient characteristics

Patient characteristics of the derivation and validation cohorts are shown in Table 1. From September 2007 to August 2008, 3,255 patients in the CNSR were eligible for the study and were included in the final analysis (Additional file 1: Figure S1 shows this in more detail). The median age was 62 (interquartile range (IQR) 53 to 72) and 61.3% were men. A total of 1,497 (46.0%) had poor functional outcome (mRS ≥3) at one year after ICH. The eligible patients were randomly divided into derivation (60%, n = 1,953) and validation (40%, n = 1,302) cohorts, which were matched with respect to baseline characteristics and one-year functional outcome after ICH (Table 1). Clinical characteristics of patients included in the study and those excluded for missing admission hematoma volume (n = 881) are listed in Additional file 1: Table S1. They were not statistically different in functional outcome at one year after ICH.

**Table 1:** Patient characteristics

**Characteristics and outcomes**	**Overall**	**Derivation cohort**	**Validation cohort**	***P *** **value**
	**(number = 3,255)**	**(number = 1,953)**	**(number = 1,302)**	
Demographics				
Age, years, median (IQR)	62 (53–72)	62 (53–72)	62 (52–72)	0.12
Gender (male), n (%)	1995 (61.3)	1189 (80.9)	806 (61.9)	0.56
Risk factors, n (%)				
Hypertension	2210 (67.9)	1338 (68.5)	872 (67.0)	0.36
Diabetes mellitus	290 (8.9)	179 (9.2)	111 (8.5)	0.53
Dyslipidemia	230 (7.1)	148 (7.6)	82 (6.3)	0.16
Atrial fibrillation	54 (1.7)	33 (1.7)	21 (1.6)	0.87
Coronary heart disease	204 (6.3)	123 (6.3)	81 (6.2)	0.93
History of stroke/TIA	889 (27.3)	543 (27.8)	346 (26.6)	0.44
Current smoker	1228 (37.7)	734 (37.6)	494 (37.9)	0.83
Heavy alcohol consumption	367 (11.3)	223 (11.4)	144 (11.1)	0.75
Transport to hospital by EMS, n (%)	1029 (31.6)	625 (32.0)	404 (31.0)	0.75
Pre-admission anticoagulation, n (%)	32 (1.0)	20 (1.0)	12 (0.9)	0.86
Pre-admission antiplatelet, n (%)	291 (8.9)	173 (8.9)	118 (9.1)	0.85
Pre-admission statin, n (%)	228 (7.0)	146 (7.5)	82 (6.3)	0.21
Admission NIHSS score, median (IQR)	9 (3–16)	9 (3–16)	9 (3–17)	0.98
Admission GCS score, median (IQR)	14 (9–15)	14 (9–15)	14 (9–15)	0.51
Admission SBP (mm Hg), median (IQR)	160 (147–180)	160 (147–180)	160 (146–180)	0.17
Admission DBP (mm Hg), median (IQR)	95 (87–106)	94 (87–105)	96 (87–108)	0.13
Admission WBC, 10^9^/L, median (IQR)	8.7 (6.7-11.3)	8.6 (6.7-11.2)	8.8 (6.7-11.6)	0.37
Admission hemoglobin, g/dL, median (IQR)	139 (126–150)	139 (126–150)	139 (127–150)	0.72
Admission platelet, 10^9^/L, median (IQR)	186 (145–230)	187 (143–232)	185 (147–227)	0.83
Admission glucose (mmol/L), median (IQR)	6.3 (5.7-7.5)	6.3 (5.7-7.5)	6.3 (5.7-7.5)	0.58
Admission creatinine (mmol/L), median (IQR)	77.0 (62.0-92.0)	77 (62–92)	76 (61–92)	0.39
Infratentorial ICH, n (%)	393 (12.1)	229 (11.7)	164 (12.6)	0.46
Hematoma volume (cm^3^), median (IQR)	12.6 (5.5-28.0)	12.8 (5.2-28.1)	12.3 (5.7-28.0)	0.93
Intraventricular extension, n (%)	962 (29.6)	587 (30.1)	375 (28.8)	0.44
Withdrawal of medical care, n (%)	404 (12.4)	247 (12.6)	157 (12.1)	0.63
Academic hospital, n (%)	1724 (53.0)	1043 (53.4)	681 (52.3)	0.55
Surgical treatment	81 (2.5)	50 (2.6)	31 (2.4)	0.82
mRS score at one year after ICH, n (%)				0.73
mRS = 0	691 (21.2)	409 (20.9)	282 (21.7)	
mRS = 1	743 (22.8)	442 (22.6)	301 (23.1)	
mRS = 2	324 (10.0)	190 (9.7)	134 (10.3)	
mRS = 3	318 (9.8)	196 (10.0)	122 (9.4)	
mRS = 4	238 (7.3)	134 (6.9)	104 (8.0)	
mRS = 5	92 (2.8)	56 (2.9)	36 (2.8)	
mRS = 6	849 (26.1)	526 (26.9)	323 (24.8)	

DBP, diastolic blood pressure; EMS, Emergency Medical System; GCS, Glasgow Coma Scale; ICH, intracerebral hemorrhage; IQR, interquartile range; mRS, modified Rankin Scale; NIHSS, National Institutes of Health Stroke Scale score; SBP, systolic blood pressure; TIA, transient ischemic attack; WBC, white blood cell count.

### Predictors of poor functional outcome at one year after ICH

The univariate analysis for potential predictors of poor functional outcome (mRS ≥3) at one year after ICH in the derivation cohort is shown in Additional file 1: Table S2 and the multivariable predictors are listed in Table 2. Age, admission NIHSS score, GCS score, blood glucose, ICH location, hematoma volume and intraventricular extension were identified as independent predictors for poor functional outcome at one year after ICH. The tolerance of covariates in the final multivariable model ranged between 0.63 and 0.99; the mean VIF was 1.36 (range: 1.08 to 1.96).

**Table 2:** Multivariable predictors of poor functional outcome (mRS ≥3) at one year after ICH in the derivation cohort (number = 1953)

**Variables**	**β-coefficients**	**SE**	**Adjusted OR**^**a**^	**95% CI**	***P *** **value**
Model intercept	−5.855				
Age (per year increase)	0.051	0.004	1.05	1.04–1.06	<0.001
Admission NIHSS score (per 1 increase)	0.092	0.006	1.10	1.08–1.11	<0.001
Admission GCS score (per 1 decrease)	0.093	0.014	1.09	1.07–1.12	<0.001
Admission blood glucose (per 1 mmol/L increase)	0.075	0.023	1.08	1.03–1.13	0.001
Infratentorial location of ICH (yes)	0.705	0.136	2.02	1.55–2.65	<0.001
Hematoma volume (per 1 cm^3^ increase)	0.018	0.002	1.02	1.01–1.03	<0.001
Intraventricular extension (yes)	0.486	0.100	1.62	1.34–1.98	<0.001

^a^Multivariable logistic regression adjusted for age, gender, stroke risk factors, transportation mode to hospital, pre-admission anticoagulation, antiplatelet and statins use, admission NIHSS score, GCS score, laboratory tests on admission (hemoglobin, white blood cell count, platelet count, blood glucose and creatinine), ICH location, hematoma volume, intraventricular extension and hospital academic status. CI, confidence interval; GCS, Glasgow Coma Scale; ICH, intracerebral hemorrhage; mRS, modified Rankin Scale; NIHSS, National Institutes of Health Stroke Scale score; OR, odds ratio; SE, standard error.

### Derivation of the ICH-FOS

**Figure 1:**
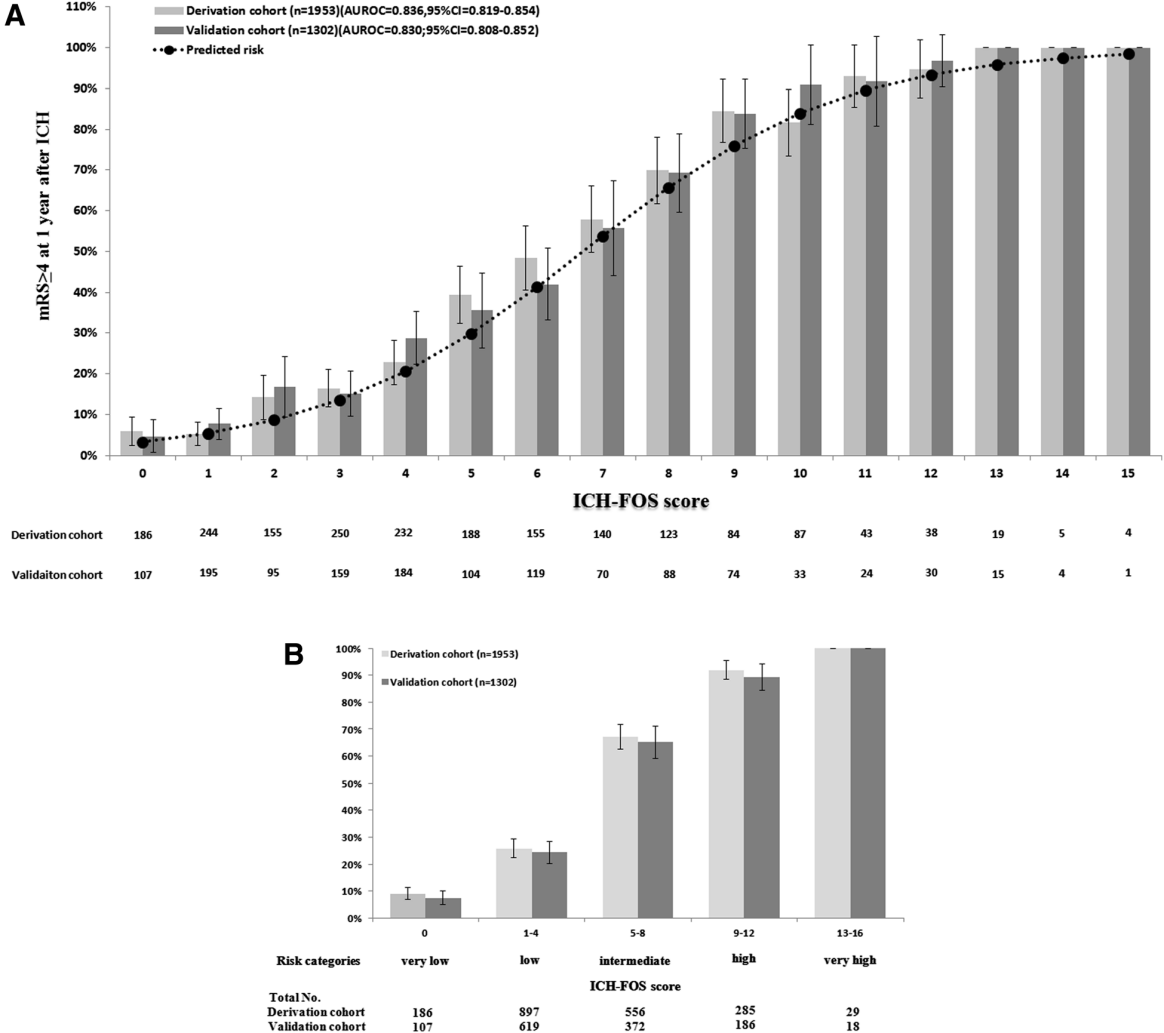
**Functional outcome at one year after ICH according to the ICH-FOS score and risk category.** Showing the proportion of poor functional outcome (mRS ≥3) at one year after ICH according to the ICH-FOS score **(A)** and risk category **(B)** in the derivation and validation cohorts, respectively. The risk of poor functional outcome increased steadily with higher ICH-FOS score. Error bars indicated 95% confidence interval of the proportion of poor functional outcome at one year after ICH for each ICH-FOS score or risk category. ICH-FOS, Intracerebral Hemorrhage Functional Outcome Score; mRS, modified Rankin Scale.

The scoring system of ICH-FOS is listed in Table 3. The median ICH-FOS was 4 (IQR: 2 to 7; range 0 to 15) in the derivation cohort. For clinical practicability, we also classified patients into five risk categories, which were assigned by four-point increments in ICH-FOS. Figure 1A and 1B show the proportion of poor functional outcome at one year after ICH according to the ICH-FOS score and risk categories in the derivation and validation cohorts, respectively. The magnitude of the ICH-FOS score had prognostic implications.

**Table 3:** Point scoring system of the ICH Functional Outcome Score (ICH-FOS)

**Items**	**Score**
**Age group**	
**≤59**	**0**
**60 to 69**	**1**
**70 to 79**	**2**
**≥80**	**4**
**Admission NIHSS score**	
**0 to 5**	**0**
**6 to 10**	**2**
**11 to 15**	**3**
**16 to 20**	**4**
**≥21**	**5**
**Admission GCS score**	
**15 to 13**	**0**
**9 to 12**	**1**
**3 to 8**	**2**
**Admission glucose (mmol/L)**	
**≤11.0**	**0**
**≥11.1**	**1**
**ICH location**	
**Supratentorial**	**0**
**Infratentorial**	**1**
**ICH volume**	
**For supratentorial location**	
**<40 ml**	**0**
**40 to 70 ml**	**2**
**>70 ml**	**2**
**For infratentorial location**	
**<10 ml**	**0**
**10 to 20 ml**	**2**
**>20 ml**	**2**
**Extension into ventricles**	
**No**	**0**
**Yes**	**1**

### Validation of the ICH-FOS

The performance of the ICH-FOS (AUROC) in the derivation and validation cohort was 0.836 (95% CI = 0.819 to 0.854) and 0.830 (95% CI = 0.808 to 0.852), respectively. Similar good discrimination was found when mRS ≥4 and mRS ≥5 was used to define poor functional outcome at one year after ICH (Additional file 1: Table S3 shows this in more detail). The Hosmer-Lemeshow test was not significant in the derivation (*P* = 0.42) and validation (*P* = 0.39) cohorts; meanwhile, the predicted and observed risk of poor functional outcome at one year after ICH was highly correlated (Additional file 1: Figure S2 shows this in more detail).

### Sensitivity analysis

We completed prespecified subgroup analyses by age, gender, hematoma location, status of medical care withdrawal and hospital academic status. Similar good discrimination was seen in these subgroups (Additional file 1: Table S4 shows this in more detail). When patients with missing admission ICH volume (n = 881) were included in analysis and coded as median ICH volume (12 cm^3^), the ICH-FOS showed good discrimination (AUROC: 0.827; 95% CI: 0.814 to 0.839) for one-year poor functional outcome (mRS ≥3) after ICH. The ICH-FOS also demonstrated good discrimination when patients lost to follow up (n = 281) were included and coded as having good functional outcome (mRS ≤2) (AUROC: 0.817; 95% CI: 0.803 to 0.832) or poor functional outcome (mRS ≥3) (AUROC: 0.822; 95% CI: 0.807 to 0.836).

### Comparative evaluation of ICH scores

**Figure 2:**
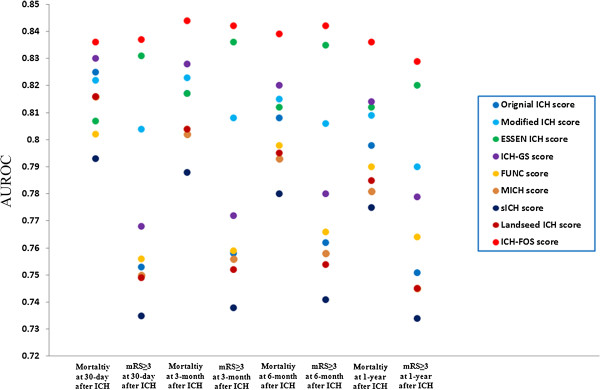
**Comparative evaluation of the ICH-FOS and existing ICH scores.** Showing the discrimination of the ICH-FOS and eight existing ICH scores with regard to poor functional outcome (mRS ≥3) and mortality at 30 days, 3 months, 6 months, and 1 year after ICH in the overall cohort (n = 3,255). ICH-FOS, Intracerebral Hemorrhage Functional Outcome Score; mRS, modified Rankin Scale.

Figure 2 shows the discrimination of the ICH-FOS and eight existing ICH scores with regard to poor functional outcome (mRS ≥3) and mortality at 30 days, 3 months, 6 months and 1 year after ICH in the overall cohort (n = 3,255) (Additional file 1: Tables S5, S6, S7, S8 show this in more detail). For 30-day poor functional outcome, AUROCs ranged from 0.735 to 0.837. The ICH-FOS and Essen ICH score had comparable AUROCs, which were significantly higher than those of other scores. For 30-day mortality, AUROCs ranged from 0.793 to 0.836. The ICH-FOS had the highest AUROC, although there was no significant pairwise difference in AUROC between ICH-FOS and the original ICH score and ICH-GS score. For three-month poor functional outcome, ICH-FOS and Essen ICH score were demonstrated to be significantly superior to other scores. For three-month mortality, six-month poor functional outcome, six-month mortality, one-year poor functional outcome and one-year mortality, the ICH-FOS consistently showed the highest AUROC and maximum Youden Index and associated PPV and NPV. The pairwise difference in AUROC between ICH-FOS and eight existing ICH scores was statistically significant (all *P* <0.001).

## Discussion

In this study, we developed and validated a risk score for predicting one-year functional outcome after ICH using information routinely available at presentation. A 16-point ICH-FOS was developed from the set of independent predictors of one-year poor functional outcome (mRS ≥3) after ICH. The ICH-FOS showed good discrimination and calibration in both the derivation and validation cohorts regardless of the specific cutpoint of mRS (mRS ≥3, ≥4, ≥5 or = 6) used to define poor outcome. This is important since different clinical care contexts or clinical research studies may define poor outcome differently. When compared to eight existing scores, the ICH-FOS showed significantly better discrimination for poor functional outcome and mortality at one year after ICH. Meanwhile, the ICH-FOS also demonstrated either comparable or significantly better discrimination for 30-day, 3-month, and 6-month poor functional outcome and mortality after ICH than eight existing scores.

To preserve the clinical utility of the model for decision-making during acute hospitalization and postdischarge, we used only patient characteristics available at presentation. We chose not to include variables related to in-hospital and postdischarge management, such as neurosurgical intervention [25,26], treatment location (in stroke unit [27] or neurocritical care unit [28,29]), withdrawal of medical care [30,31] and rehabilitation, despite the fact that these factors might influence functional outcome at one year after ICH. This model, therefore, predicts the expected outcome at one year after ICH at presentation.

During the past decade, several prognostic models have been developed for ICH. In 2001, Hemphill *et al*. [6] introduced the original ICH score (oICH), which is one of the first simple and easily assessable clinical grading scales for ICH. Since then, a number of modifications to oICH [7,8] and other pragmatic ICH scores [7,9-16] have been proposed. Although some of these ICH scores have been internally or externally validated, none of them has been universally accepted and consistently used in routine clinical practice and clinical research [5]. For a clinical grading scale to become widely used and effective, it must be reliable, accurate and practical. The ICH-FOS is different from the compared ICH scores in several aspects: First, for reliability, the ICH-FOS was developed based on the largest derivation (n = 1,953) and validation (n = 1,302) cohorts, which included consecutive ICH patients who were outside of clinical trial and were more representative of real-world clinical practice. Additionally, sensitivity analysis showed that the ICH-FOS was robust against medical care withdrawal and was effective for ICH patients of different ages, gender and hematoma location. Second, for accuracy, the ICH-FOS demonstrated good discrimination and calibration with regard to one-year poor functional outcome and mortality after ICH in the derivation and validation cohorts. Finally, for practicality, the ICH-FOS consists of factors that are readily available at presentation. In addition, by a simple score, patients can be easily stratified into five risk categories, which might be useful for both routine clinical practice and clinical research.

With several ICH related risk-stratification and prognostic models available, identification of the most accurate and reliable grading scale(s) would be of great value to patients, clinicians, and researchers. In this study, we compared the discrimination of the ICH-FOS and eight existing ICH scores with regard to both poor functional outcome (mRS ≥3) and mortality at 30 days, 3 months, 6 months, and 1 year after ICH in a large cohort. For one-year poor functional outcome and mortality, the ICH-FOS was shown to be significantly better than eight prior ICH scores. Meanwhile, the ICH-FOS also demonstrated either comparable or significantly better discrimination for ICH outcomes at 30 days, 3 months and 6 months after ICH. Although promising, caution has to be taken when interpreting the results: first, the study populations for derivation and validation of these ICH scores are different. The baseline characteristics of our study were different from those of western cohorts used to develop prior ICH scores [6,9-11], such as younger age of ICH onset, less severity of neurological deficit, smaller hematoma volume on admission and fewer intraventricular extensions in our study. It is not our primary aim to compare the differences of these ICH cohorts and it is hard to explain the reasons due to differences in study design and study population. Second, there might be complex genetic, social, cultural, and economic factors as well as regional management philosophies and preferences that are difficult to account for when grading scales are developed or applied to a distinct population. Finally, the intended outcome (functional outcome versus mortality), outcome assessent methods (glasgow outcomes scale GOS versus barthel index BI versus mRS), and timing of follow-up (in-hospital versus 30-day versus 3-month versus 6-month versus 1-year after ICH) are different for these existing ICH scores. In the future, the standardization of these variables would greatly facilitate validation and comparison of ICH grading scales [5].

Despite advances in medical knowledge, treatment of ICH remains strictly supportive with not many evidence-based interventions currently available. This might be at least partially due to inclusion of patients with unbalanced, too high, or too low risk of developing poor functional outcome and mortality in prior studies. Although there was a statistically significant difference, our study showed that the ICH-FOS and several other ICH predictive tools, such as the Essen ICH score, the ICH-GS score and the original ICH score were clinically useful in stratifying patients according to the potential risk of poor functional outcome or mortality. Thus, clinicians and clinical researchers have a great deal of flexibility in their choice of ICH predictive rules based on their familiarity and preference. It is expected that randomized controlled ICH efficacy trials with stratification of patients’ potential long-term prognosis will be conducted in the future.

Our study has limitations that deserve comment. First, like all observational studies, we cannot rule out the possibility that additional variables (unmeasured confounders) might be predictive of long-term functional outcome after ICH. In the future, novel biochemical [32], neuroimaging [33] or genetic markers might be identified and would improve the discrimination of our model. Second, admission blood glucose and hematoma volume were associated with one-year functional outcome after ICH in a continuous manner. However, for clinical practicability, they were categorized and this inevitably led to loss of information. Furthermore, it is possible that the cutoff value may vary with the development of our knowledge of ICH pathophysiology, especially for blood glucose. Third, Intraventricular hemorrhage (IVH) was dichotomized into present or absent as opposed to the use of IVH volume. Fourth, due to the fact that Graeb score and admission body temperature were not routinely collected in the CNSR, we cannot externally validate two prior ICH scores (the mICHs [8] and new ICH score [7]) in this study. Another two ICH scores were also excluded because they focused on a specific subgroup of patients (ICH patients with hemodialysis [13] and known hypertension [16]). Finally, both the derivation and validation cohorts originated from an Asian population and the ICH-FOS needs to be further validated in different populations.

## Conclusions

The ICH-FOS is a valid clinical grading scale for predicting functional outcome at one year after ICH. Further studies to validate the ICH-FOS in different populations are needed.

## Key messages

• Age, admission NIHSS score, GCS score, blood glucose, ICH location, hematoma volume and intraventricular extension were identified as independent predictors for poor functional outcome (mRS ≥3) at one year after ICH.

• A 16-point ICH-FOS was developed from these independent predictors of one-year poor functional outcome (mRS ≥3) after ICH.

• The ICH-FOS showed good discrimination and calibration in large derivation (n = 1,953) and validation (n = 1,302) cohorts.

• The ICH-FOS also demonstrated comparable or significantly better discrimination with regard to 30-day, 3-month and 6-month poor functional outcome (mRS ≥3) and mortality when compared to eight existing ICH scores.

• Further validation of the ICH-FOS in different populations is needed.

## Abbreviations

AUROC: Area under the receiver operating characteristic curve; BI: Barthel index; CI: Confidence interval; CNSR: China National Stroke Registry; DBP: Diastolic blood pressure; EMS: Emergency medical system; GCS: Glasgow coma scale; GOS: Glasgow outcome scale; ICH: Intracerebral hemorrhage; ICH-FOS: ICH functional outcome score; IQR: Interquartile range; IVH: Intraventricular hemorrhage; mRS: Modified rankin scale; NIHSS: National Institutes of Health Stroke Scale score; NPV: Negative predictive value; OR: Odds ratio; PPV: Positive predict value; SBP: Systolic blood pressure; SE: Standard error; TIA: Transient ischemic attack; VIF: Variance inflation factor; WBC: White cell count.

## Competing interests

The authors declare that they have no completing interests.

## Authors’ contributions

RJ and YW (Yongjun Wang) conceived of the study, participated in its design and drafted the manuscript. HS and RJ carried out statistical analysis. RJ, HS, YP, PW, GL, YW (Yilong Wang), HL, XZ, and YW (Yongjun Wang) participated in analysis or interpretation of data, and revised the manuscript for important intellectual content. All authors read and approved the final manuscript.

## Supplementary Material

Additional file 1: Table S1 Clinical characteristics of patients included in the study and those excluded for missing admission hematoma volume (n = 881). **Table S2.** Univariate predictor of poor functional outcome (mRS ≥3) at one year after ICH in the derivation cohort (n = 1,953). **Table S3.** Discrimination of the ICH-FOS for poor functional outcome at one year after ICH in the derivation and validation cohorts. **Table S4.** Subgroup analysis of discrimination of the ICH-FOS for one-year poor functional outcome (mRS ≥3) after ICH. **Table S5.** Discrimination of the ICH-FOS and eight prior ICH scores for poor functional outcome (mRS ≥3) and mortality at 30 days after ICH (n = 3,255). **Table S6.** Discrimination of the ICH-FOS and eight prior ICH scores for poor functional outcome (mRS ≥3) and mortality at three months after ICH (n = 3,255). **Table S7.** Discrimination of the ICH-FOS and eight prior ICH scores for poor functional outcome (mRS ≥3) and mortality at six months after ICH (n = 3,255). **Table S8.** Discrimination of the ICH-FOS and eight prior ICH scores for poor functional outcome (mRS ≥3) and mortality at one year after ICH (n = 3,255). **Figure S1.** Patient flowchart. **Figure S2.** Plot of observed versus predicted risk of poor functional outcome (mRS ≥3) at one year after ICH in the derivation and validation cohorts. **Appendix A.** The CNSR investigators. **Appendix B.** Institutional Review Board within the CNSR network. Click here for file
