# Supplementary material for: A novel risk score to predict 1-year functional outcome after intracerebral hemorrhage and comparison with existing scores
Source: Crit Care. 2013 Nov 29;17(6):R275. doi: 10.1186/cc13130 (PMC4056008; doi:10.1186/cc13130)
Supplement: Additional file 1: Table S1 — Clinical characteristics of patients included in the study and those excluded for missing admission hematoma volume (n = 881). Table S2. Univariate predictor of poor functional outcome (mRS ≥3) at one year after ICH in the derivation cohort (n = 1,953). Table S3. Discrimination of the ICH-FOS for poor functional outcome at one year after ICH in the derivation and validation cohorts. Table S4. Subgroup analysis of discrimination of the ICH-FOS for one-year poor functional outcome (mRS ≥3) after ICH. Table S5. Discrimination of the ICH-FOS and eight prior ICH scores for poor functional outcome (mRS ≥3) and mortality at 30 days after ICH (n = 3,255). Table S6. Discrimination of the ICH-FOS and eight prior ICH scores for poor functional outcome (mRS ≥3) and mortality at three months after ICH (n = 3,255). Table S7. Discrimination of the ICH-FOS and eight prior ICH scores for poor functional outcome (mRS ≥3) and mortality at six months after ICH (n = 3,255). Table S8. Discrimination of the ICH-FOS and eight prior ICH scores for poor functional outcome (mRS ≥3) and mortality at one year after ICH (n = 3,255). Figure S1. Patient flowchart. Figure S2. Plot of observed versus predicted risk of poor functional outcome (mRS ≥3) at one year after ICH in the derivation and validation cohorts. Appendix A. The CNSR investigators. Appendix B. Institutional Review Board within the CNSR network. [file cc13130-S1.pdf]

Additional table 1. Clinical characteristics of patients included in the study and those excluded for missing admission hematoma volume (n=881)

	Included patients (n=3255)	Exclude patients for missing ICH volume (n=881)	P value
Demographics			
Age, y, median (IQR)	62 (53-72)	60 (51-72)	<0.001
Gender (male), n (%)	1995 (61.3)	554 (62.9)	0.41
Risk factors, n (%)			
Hypertension	2210 (67.9)	593 (67.3)	0.75
Diabetes mellitus	290 (8.9)	98 (11.1)	0.05
Dyslipidemia	230 (7.1)	44 (5.0)	0.03
Atrial fibrillation	54 (1.7)	22 (2.5)	0.12
Coronary heart disease	204 (6.3)	71 (8.1)	0.07
History of stroke/TIA	889 (27.3)	231 (26.2)	0.83
Current Smoker	1228 (37.7)	316 (36.2)	0.43
Heavy alcohol consumption	367 (11.3)	130 (15.5)	<0.001
Transport to hospital by EMS, n (%)	1029 (31.6)	274 (31.1)	0.95
Pre-admission anticoagulation, n (%)	32 (1.0)	7 (0.9)	1.00
Admission NIHSS score, median (IQR)	9 (3-16)	8 (2-19)	0.21
Admission GCS score, median (IQR)	14 (9-15)	14 (8-15)	0.02
Admission SBP (mm Hg), median (IQR)	160 (147-180)	160 (144-180)	0.10
Admission DBP (mm Hg), median (IQR)	95 (87-106)	92 (82-103)	0.001
Admission WBC count, 10^9/L, median (IQR)	8.7 (6.7-11.3)	9.6 (7.2-12.9)	<0.001
Admission hemoglobin, g/dL, median (IQR)	139 (126-150)	138 (127-151)	0.47
Admission platelet, 10^9/L, median (IQR)	186 (145-230)	198 (147-249)	<0.001
Admission glucose (mmol/L), median (IQR)	6.3 (5.7-7.5)	6.6 (5.9-8.2)	<0.001
Admission creatinine (mmol/L), median (IQR)	77.0 (62.0-92.0)	74.6 (60.6-90.0)	0.14
Infratentorial location, n (%)	393 (12.1)	91 (10.3)	0.15
Intraventricular extension, n (%)	962 (29.6)	195 (22.1)	<0.001
Withdrawal of medical care, n (%)	404 (12.4)	129 (14.6)	<0.001
Functional outcome at 1 year after ICH, n (%)			
mRS≥3	1497 (46.0)	389 (44.2)	0.34
mRS≥4	1179 (36.2)	309 (35.1)	0.55
mRS≥5	941 (28.9)	231 (26.2)	0.12

Abbreviation: IQR, Interquartile Range; TIA, Transient Ischemic Attack; EMS, Emergency Medical System; NIHSS, National Institutes of Health Stroke Scale score; GCS, Glasgow Coma Scale; SBP, Systolic Blood Pressure; DBP, Diastolic Blood Pressure; WBC, White Cell Count; ICH, Intracerebral Hemorrhage; mRS, modified Rankin Scale.

Additional table 2. Univariate predictor of poor functional outcome (mRS$\geq$3) at 1 year after ICH in the derivation cohort (n=1953)

	**Increment/categories**	**OR**	**95% C.I.**	**P value**
Demographics				
Age, year	1 year increase	1.04	1.03-1.05	<0.001
Gender	female vs. male	1.19	1.03-1.37	0.02
Risk factors				
Hypertension	yes vs. no	1.01	0.87-1.17	0.90
Diabetes mellitus	yes vs. no	1.45	1.14-1.85	0.002
Dyslipidemia	yes vs. no	0.73	0.55-0.95	0.02
Atrial fibrillation	yes vs. no	1.86	1.07-3.23	0.03
Coronary heart disease	yes vs. no	1.32	0.99-1.75	0.06
History of stroke/TIA	yes vs. no	1.58	1.31-1.92	<0.001
Current Smoker	yes vs. no	0.85	0.73-0.98	0.03
Heavy alcohol consumption	yes vs. no	0.72	0.58-0.90	0.004
Transport to hospital by EMS	yes vs. no	2.83	2.43-3.29	<0.001
Pre-admission anticoagulation	yes vs. no	1.18	0.86-2.12	0.42
Pre-admission antiplatelet	yes vs. no	1.23	0.97-1.58	0.11
Pre-admission statins	yes vs. no	0.70	0.56-0.96	0.02
Admission NIHSS score	1 point increase	1.13	1.12-1.14	<0.001
Admission GCS score	1 point decrease	1.28	1.26-1.31	<0.001
Admission SBP (mmHg)	10 mm Hg increase	1.09	1.06-1.11	<0.001
Admission DBP (mmHg)	10 mm Hg increase	1.03	0.99-1.07	0.19
Admission WBC count	1×10^9/L increase	1.00	0.99-1.00	0.58
Admission hemoglobin	1×g/L increase	0.99	0.99-1.00	0.26
Admission platelet	1×10^9/L increase	1.00	0.99-1.00	0.42
Admission glucose	1×mmol/L increase	1.23	1.18-1.27	<0.001
Admission creatinine	1×mmol/L increase	1.00	1.00-1.01	0.002
ICH location	Infratentorial vs. supratentorial	1.34	1.09-1.66	0.007
Hematoma volume	$1\times cm^3$ increase	1.03	1.02-1.04	<0.001
Intraventricular extension	yes vs. no	2.87	2.46-3.36	<0.001
Withdrawal of medical care	yes vs. no	3.28	2.62-4.12	<0.001
Hospital academic status	academic vs. non-academic	0.82	0.72-1.05	0.10
Surgical treatment	surgical vs. medical treatment	1.56	0.88-2.63	0.08

Abbreviation: OR, Odds Ratio; C.I., Confidence Interval; TIA, Transient Ischemic Attack; EMS, Emergency Medical System; NIHSS, National Institutes of Health Stroke Scale score; GCS, Glasgow Coma Scale; SBP, Systolic Blood Pressure; DBP, Diastolic Blood Pressure; WBC, White Cell Count; ICH, Intracerebral Hemorrhage.

Additional table 3. Discrimination of the ICH-FOS for poor functional outcome at 1 year after ICH in the derivation and validation cohort

	AUROC	95% C.I.	P value	Youden Index	Cutoff	Sensitivity	Specificity	PPV	NPV
In the derivation cohort (n=1953)									
mRS≥3 at 1 year after ICH	0.836	0.819-0.854	<0.0001	0.530	5	0.728	0.802	0.763	0.771
mRS≥4 at 1 year after ICH	0.849	0.831-0.866	<0.0001	0.624	5	0.805	0.819	0.721	0.819
mRS≥5 at 1 year after ICH	0.840	0.823-0.856	<0.0001	0.536	6	0.734	0.802	0.612	0.876
mRS=6 at 1 year after ICH	0.831	0.813-0.851	<0.0001	0.522	6	0.743	0.779	0.553	0.892
In the validation cohort (n=1302)									
mRS≥3 at 1 year after ICH	0.830	0.808-0.852	<0.0001	0.519	5	0.728	0.791	0.740	0.781
mRS≥4 at 1 year after ICH	0.836	0.813-0.859	<0.0001	0.613	5	0.796	0.815	0.706	0.879
mRS≥5 at 1 year after ICH	0.839	0.818-0.859	<0.0001	0.514	6	0.724	0.790	0.568	0.883
mRS=6 at 1 year after ICH	0.843	0.822-0.863	<0.0001	0.552	6	0.759	0.793	0.547	0.909

Abbreviation；ICH, Intracerebral Hemorrhage; AUROC, Area Under the Receiver Operating Characteristic Curve; C.I., Confidence Interval; PPV, Positive Predictive Value; NPV, Negative Predictive Value.

Additional table 4. Subgroup analysis of discrimination of the ICH-FOS for 1-year poor functional outcome (mRS≥3) after ICH

	**Derivation cohort (n=1953)**		**Validation cohort (n=1302)**	
	**AUROC**	**95% C.I.**	**AUROC**	**95% C.I.**
Overall cohort	0.836	0.819-0.854	0.830	0.808-0.852
Subgroups				
Age				
≤59	0.814	0.783-0.845	0.800	0.768-0.834
≥60	0.835	0.812-0.858	0.843	0.814-0.872
Gender				
Male	0.850	0.828-0.872	0.845	0.818-0.872
Female	0.813	0.783-0.843	0.807	0.769-0.845
Hematoma location				
Supratentorial	0.837	0.818-0.856	0.827	0.803-0.851
Infratentorial	0.828	0.776-0.881	0.848	0.788-0.908
Withdrawal of medical care				
No	0.828	0.809-0.848	0.826	0.801-0.850
Yes	0.858	0.812-0.905	0.826	0.762-0.890
Hospital academic status				
Academic	0.844	0.820-0.868	0.841	0.810-0.872
Non-academic	0.829	0.802-0.855	0.821	0.800-0.853

Abbreviation: AUROC, Area Under the Receiver Operating Characteristic Curve; C.I., Confidence Interval.

Additional table 5. Discrimination of the ICH-FOS and 8 prior ICH scores for poor functional outcome (mRS≥3) and mortality at 30-day after ICH (n=3255)

	AUROC	95% CI	Δ AUROC*	P value[&]	Youden Index	Cutoff	Sensitivity	Specificity	PPV	NPV
**mRS≥3 at 30-day after ICH**										
Original ICH score (2001)	0.753	0.738-0.768	0.084	<0.0001	0.378	2	0.526	0.852	0.778	0.645
Modified ICH score (2003)	0.804	0.790-0.818	0.033	<0.0001	0.462	2	0.611	0.851	0.802	0.688
Essen ICH score (2006)	0.831	0.817-0.844	0.006	0.133	0.523	3	0.755	0.768	0.763	0.760
ICH-GS score (2007)	0.768	0.753-0.782	0.069	<0.0001	0.430	8	0.660	0.770	0.740	0.696
FUNC score (2008)	0.756	0.741-0.771	0.081	<0.0001	0.406	7	0.593	0.813	0.759	0.669
MICH score (2008)	0.750	0.735-0.765	0.087	<0.0001	0.378	2	0.542	0.836	0.766	0.649
sICH score (2009)	0.735	0.719-0.750	0.102	<0.0001	0.418	7	0.623	0.795	0.750	0.681
Landseed ICH score (2011)	0.749	0.732-0.763	0.088	<0.0001	0.375	1	0.783	0.592	0.655	0.734
ICH-FOS score (2012)	0.837	0.824-0.894	Ref	-	0.532	5	0.712	0.820	0.796	0.742
**Mortality at 30-day after ICH**										
Original ICH score (2001)	0.825	0.811-0.838	0.011	0.169	0.498	2	0.753	0.745	0.363	0.940
Modified ICH score (2003)	0.822	0.808-0.835	0.014	0.036	0.494	2	0.793	0.701	0.339	0.946
Essen ICH score (2006)	0.807	0.793-0.820	0.029	<0.0001	0.513	4	0.774	0.739	0.364	0.944
ICH-GS score (2007)	0.830	0.817-0.843	0.006	0.396	0.513	9	0.691	0.822	0.428	0.932
FUNC score (2008)	0.802	0.788-0.816	0.034	<0.0001	0.480	6	0.624	0.856	0.455	0.922
MICH score (2008)	0.816	0.802-0.829	0.02	0.029	0.508	2	0.778	0.730	0.358	0.945
sICH score (2009)	0.793	0.779-0.807	0.043	<0.0001	0.485	7	0.820	0.665	0.321	0.950
Landseed ICH score (2011)	0.816	0.802-0.829	0.02	0.029	0.509	2	0.740	0.769	0.382	0.939
ICH-FOS score (2012)	0.836	0.822-0.848	Ref	-	0.523	7	0.710	0.813	0.424	0.936

Abbreviation; AUROC, Area Under the Receiver Operating Characteristic Curve; CI, Confidential Interval; PPV, Positive Predictive Value; NPV, Negative Predictive Value.

*ΔAUROC denoted the difference in AUROC between Original ICH score and compared ICH scores for outcome at hospital discharge.

[&] P value of comparing pairwise AUROCs with Delong's method.

Additional table 6. Discrimination of the ICH-FOS and 8 prior ICH scores for poor functional outcome (mRS≥3) and mortality at 3-month after ICH (n=3255)

	AUROC	95% CI	Δ AUROC*	P value[&]	Youden Index	Cutoff	Sensitivity	Specificity	PPV	NPV
**mRS≥3 at 3-month after ICH**										
Original ICH score (2001)	0.758	0.743-0.772	0.084	<0.0001	0.385	2	0.532	0.853	0.778	0.654
Modified ICH score (2003)	0.808	0.794-0.821	0.034	<0.0001	0.472	2	0.619	0.853	0.802	0.698
Essen ICH score (2006)	0.836	0.823-0.848	0.006	0.122	0.531	3	0.762	0.769	0.761	0.770
ICH-GS score (2007)	0.772	0.757-0.786	0.07	<0.0001	0.435	8	0.665	0.770	0.736	0.704
FUNC score (2008)	0.759	0.744-0.773	0.083	<0.0001	0.41	7	0.597	0.813	0.755	0.676
MICH score (2008)	0.756	0.741-0.770	0.086	<0.0001	0.387	2	0.549	0.838	0.765	0.658
sICH score (2009)	0.738	0.723-0.753	0.104	<0.0001	0.423	7	0.629	0.794	0.747	0.789
Landseed ICH score (2011)	0.752	0.737-0.767	0.09	<0.0001	0.382	1	0.789	0.593	0.652	0.745
ICH-FOS score (2012)	0.842	0.829-0.854	Ref	-	0.543	5	0.721	0.822	0.796	0.753
**mortality at 3-month after ICH**										
Original ICH score (2001)	0.817	0.803-0.830	0.027	<0.0001	0.480	2	0.720	0.760	0.428	0.916
Modified ICH score (2003)	0.823	0.810-0.836	0.021	<0.0001	0.501	2	0.780	0.721	0.411	0.929
Essen ICH score (2006)	0.817	0.803-0.830	0.027	<0.0001	0.516	4	0.757	0.759	0.439	0.926
ICH-GS score (2007)	0.828	0.814-0.840	0.016	<0.0001	0.502	9	0.663	0.839	0.507	0.909
FUNC score (2008)	0.804	0.790-0.817	0.04	<0.0001	0.470	6	0.598	0.872	0.538	0.897
MICH score (2008)	0.802	0.788-0.816	0.042	<0.0001	0.481	2	0.737	0.744	0.418	0.919
sICH score (2009)	0.788	0.774-0.802	0.056	<0.0001	0.485	7	0.802	0.683	0.387	0.932
Landseed ICH score (2011)	0.804	0.790-0.818	0.04	<0.0001	0.482	2	0.699	0.783	0.445	0.912
ICH-FOS score (2012)	0.844	0.832-0.857	Ref	-	0.538	6	0.786	0.752	0.442	0.934

Abbreviation； AUROC, Area Under the Receiver Operating Characteristic Curve; CI, Confidential Interval; PPV, Positive Predictive Value; NPV, Negative Predictive Value.

*Δ AUROC denoted the difference in AUROC between original ICH score and compared ICH scores for outcome at 3-month after ICH.

[&] P value of comparing pairwise AUROCs with Delong's method.

Additional table 7. Discrimination of the ICH-FOS and 8 prior ICH scores for poor functional outcome (mRS≥3) and mortality at 6-month after ICH (n=3255)

	AUROC	95% CI	Δ AUROC*	P value&	Youden Index	Cutoff	Sensitivity	Specificity	PPV	NPV
**mRS≥3 at 6-month after ICH**										
Original ICH score (2001)	0.762	0.747-0.777	0.08	<0.0001	0.389	2	0.542	0.847	0.759	0.675
Modified ICH score (2003)	0.806	0.792-0.819	0.036	<0.0001	0.471	2	0.628	0.843	0.780	0.718
Essen ICH score (2006)	0.825	0.821-0.847	0.017	<0.0001	0.525	3	0.770	0.755	0.736	0.786
ICH-GS score (2007)	0.780	0.766-0.795	0.062	<0.0001	0.443	8	0.678	0.765	0.720	0.728
FUNC score (2008)	0.766	0.751-0.780	0.076	<0.0001	0.418	7	0.610	0.808	0.738	0.699
MICH score (2008)	0.758	0.742-0.772	0.084	<0.0001	0.388	2	0.557	0.831	0.745	0.679
sICH score (2009)	0.741	0.726-0.756	0.101	<0.0001	0.427	7	0.639	0.788	0.728	0.710
Landseed ICH score (2011)	0.754	0.739-0.769	0.088	<0.0001	0.384	1	0.798	0.586	0.632	0.766
ICH-FOS score (2012)	0.842	0.829-0.854	Ref	-	0.543	5	0.732	0.811	0.775	0.773
**mortality at 6-month after ICH**										
Original ICH score (2001)	0.808	0.794-0.821	0.031	<0.0001	0.463	2	0.695	0.768	0.465	0.897
Modified ICH score (2003)	0.815	0.801-0.828	0.024	<0.0001	0.488	2	0.757	0.731	0.449	0.912
Essen ICH score (2006)	0.812	0.799-0.826	0.027	<0.0001	0.508	4	0.738	0.770	0.482	0.910
ICH-GS score (2007)	0.820	0.807-0.833	0.019	0.0006	0.491	9	0.642	0.849	0.553	0.891
FUNC score (2008)	0.798	0.784-0.812	0.041	<0.0001	0.456	7	0.742	0.714	0.429	0.905
MICH score (2008)	0.793	0.779-0.807	0.046	<0.0001	0.466	2	0.713	0.753	0.455	0.900
sICH score (2009)	0.780	0.765-0.794	0.059	<0.0001	0.473	7	0.780	0.693	0.425	0.916
Landseed ICH score (2011)	0.795	0.781-0.809	0.044	<0.0001	0.465	2	0.674	0.791	0.483	0.893
ICH-FOS score (2012)	0.839	0.826-0.852	Ref	-	0.538	6	0.772	0.766	0.489	0.920

Abbreviation； AUROC, Area Under the Receiver Operating Characteristic Curve; CI, Confidential Interval; PPV, Positive Predictive Value; NPV, Negative Predictive Value.

*Δ AUROC denoted the difference in AUROC between original ICH score and compared ICH scores for outcome at 3-month after ICH.

& P value of comparing pairwise AUROCs with Delong's method.

Additional table 8. Discrimination of the ICH-FOS and 8 prior ICH scores for poor functional outcome (mRS≥3) and mortality at 1-year after ICH (n=3255)

	AUROC	95% CI	Δ AUROC*	P value[&]	Youden Index	Cutoff	Sensitivity	Specificity	PPV	NPV
**mRS≥3 at 12-month after ICH**										
Original ICH score (2001)	0.751	0.736-0.766	0.078	<0.0001	0.378	2	0.540	0.838	0.739	0.682
Modified ICH score (2003)	0.790	0.775-0.804	0.039	<0.0001	0.447	2	0.620	0.827	0.753	0.719
Essen ICH score (2006)	0.810	0.806-0.833	0.019	<0.0001	0.498	3	0.613	0.885	0.819	0.728
ICH-GS score (2007)	0.779	0.764-0.793	0.05	<0.0001	0.434	8	0.678	0.756	0.703	0.734
FUNC score (2008)	0.764	0.750-0.779	0.065	<0.0001	0.418	7	0.610	0.808	0.696	0.707
MICH score (2008)	0.745	0.730-0.760	0.084	<0.0001	0.371	2	0.552	0.819	0.722	0.682
sICH score (2009)	0.734	0.718-0.749	0.095	<0.0001	0.415	7	0.637	0.778	0.709	0.716
Landseed ICH score (2011)	0.745	0.729-0.760	0.084	<0.0001	0.098	2	0.512	0.586	0.752	0.673
ICH-FOS score (2012)	0.829	0.815-0.842	Ref	-	0.513	5	0.722	0.791	0.747	0.770
**mortality at 12-month after ICH**										
Original ICH score (2001)	0.798	0.783-0.811	0.038	<0.0001	0.449	2	0.668	0.781	0.518	0.870
Modified ICH score (2003)	0.809	0.795-0.822	0.027	<0.0001	0.477	2	0.731	0.746	0.504	0.887
Essen ICH score (2006)	0.812	0.798-0.825	0.024	<0.0001	0.507	4	0.719	0.788	0.545	0.888
ICH-GS score (2007)	0.814	0.800-0.827	0.022	0.0001	0.478	8	0.797	0.681	0.469	0.905
FUNC score (2008)	0.790	0.775-0.804	0.046	<0.0001	0.452	7	0.723	0.729	0.485	0.882
MICH score (2008)	0.781	0.767-0.796	0.055	<0.0001	0.445	2	0.681	0.764	0.504	0.872
sICH score (2009)	0.775	0.760-0.789	0.061	<0.0001	0.469	7	0.760	0.709	0.480	0.893
Landseed ICH score (2011)	0.785	0.771-0.799	0.051	<0.0001	0.447	2	0.644	0.803	0.536	0.865
ICH-FOS score (2012)	0.836	0.822-0.848	Ref	-	0.523	6	0.742	0.781	0.545	0.896

Abbreviation； AUROC, Area Under the Receiver Operating Characteristic Curve; CI, Confidential Interval; PPV, Positive Predictive Value; NPV, Negative Predictive Value.

*Δ AUROC denoted the difference in AUROC between original ICH score and compared ICH scores for outcome at 3-month after ICH.

[&] P value of comparing pairwise AUROCs with Delong's method.

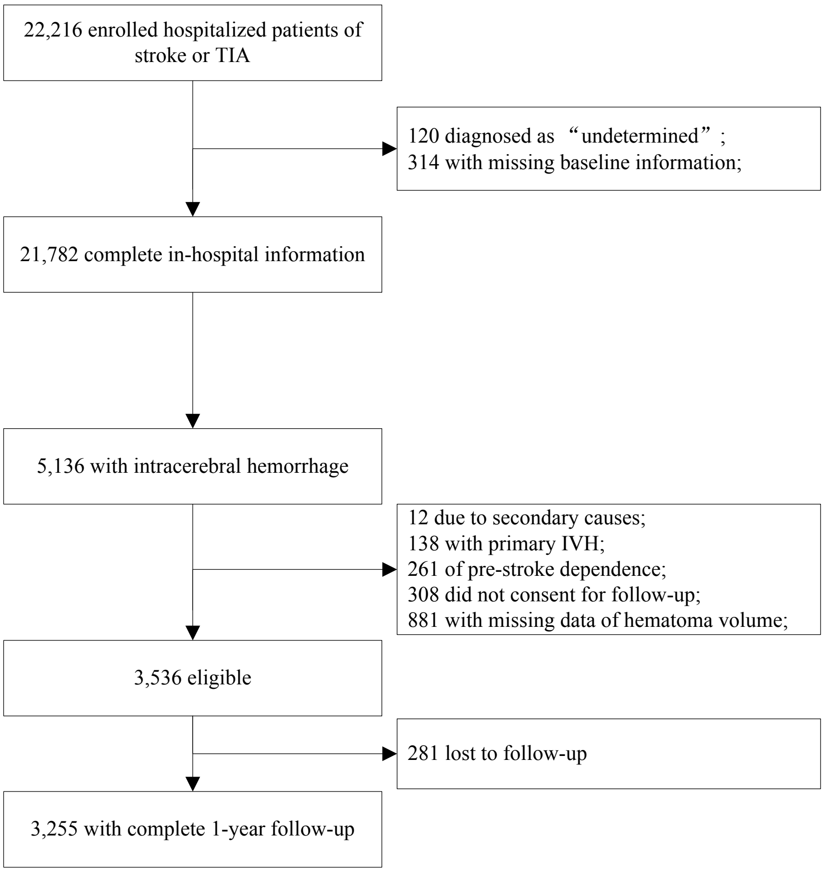

Additional figure 1. Patient flowchart

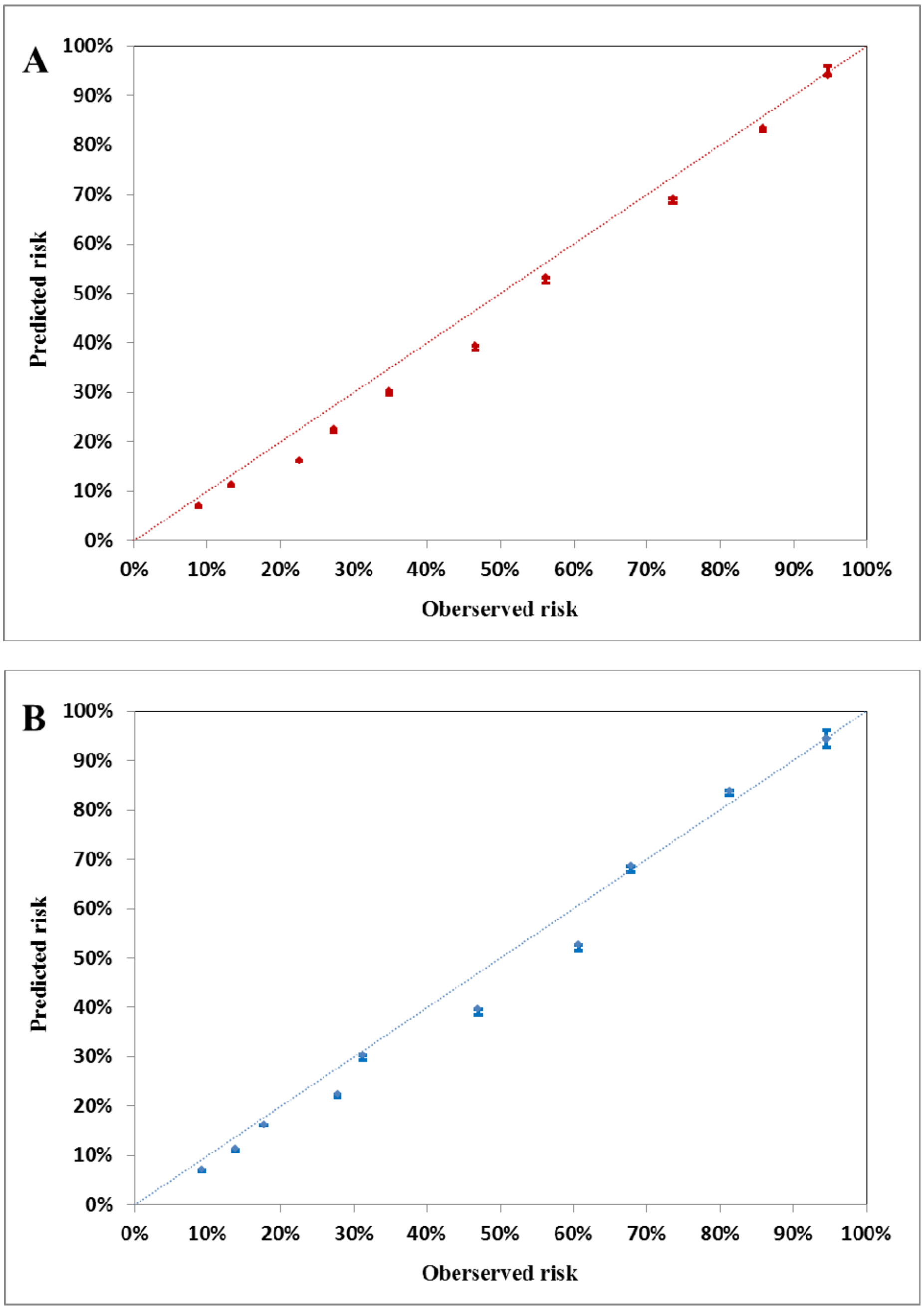

Additional figure 2. Plot of observed versus predicted risk of poor functional outcome (mRS≥3) at 1 year after ICH in the derivation and validation cohorts

Additional figure legend

Plot of observed versus predicted risk of poor functional outcome (mRS≥3) at 1 year after ICH with 95% confidence interval (C.I.) in the derivation and validation cohorts according to 10 deciles of predicted risk. Overall, there was a very high correlation between observed and predicted risk in the derivation cohort (A) (n=1953; r=0.995, P<0.001) and validation cohort (B) (n=1302; r=0.994, P<0.001), which indicated excellent calibration.

Appendix A: The CNSR investigators

Yongjun Wang, Beijing Tiantan Hospital; Qi Bi, Beijing Anzhen Hospital; Weiwei Zhang, Beijing Military District Gengral hospital of Chinese People's Liberation Army; Liying Cui, Peking Union Medical College Hospital of Peking University; Yuheng Sun, Beijing Jishuitan Hospital; Maolin He, Beijing Shijitan Hospital; Dongsheng Fan, Peking University Third Hospital; Xunming Ji, Beijing Xuanwu Hospital; Jimei Li, Beijing Friendship Hospital Affiliated to Capital Medical University; Fang Zhang, Beijing Guangwai Hospital; Kai Feng, Beijing Shunyi District Hospital; Xiaojun Zhang, Beijing Tongren Hospital; Yansheng Li, Shanghai Renji Hospital; Shaoshi Wang, Shanghai First Municipal People's Branch hospital; Wei Fan, Zhongshan Hospital of Fudan University; Zhenguo Liu, Xin Hua Hospital Affiliated to Shanghai Jiao Tong University; Xiaojiang Sun, The sixth People'sHospital Affiliated to Shanghai JiaoTong University; Wei Li, Shanghai Ninth People's Hospital Affiliated to Shanghai JiaoTong University; Jianrong Liu, ShanghaiRuijin Hospital; Xu Chen, Shanghai 8th People's Hospital; Qingke Bai, Pudong New Area People's Hospital; Dexiang Gu, Shanghai Yangpu Area Shidong Hospital; Xin Li, Shanghai Yangpu Area Center Hospital; Qiang Dong, Huashan Hospital of Fudan University; Yan Cheng, Tianjin Medical University Gengeal Hospital; Lan Yu, Tianjin Huanhu Hospital; Bin Li, Dagang Oilfield Gengeal Hospital; Tongyu Wang, Bohai Oilfield Hospital; Kun Zhao, Baodi District People's Hospital of Tianjin; Chaodong Zhang, The First Affiliated Hospital of China Medical University; Dingbo Tao, The First Afflicated Hospital of Dlian Medical University; Lin Yin, The Second Affiliated Hospital of Dlian Medical University; Fang Qu, Dlian Second People's hospital; Jingbo Zhang, Dlian Third People's hospital; Jianfeng Wang, Dalian Central hospital; Ying Lian, Dalian Economic and Technological Development District Hospital; Fang Qu, Shenying Military District General hospital of Chinese People's Liberation Army; Jun Fan, Shenyang Military District 202 Hospital; Ying Gao, National Traditional Chinese Medicine (TCM)Thrombus Treatment Center of Liaoning Province; Mingdong Cheng, En'liang hopital of Tai'an County; Jiang Wu, The First Clinical College of Jilin University; Huashan Sun, Jilin Chemical Industrial Group General hopital; Jinying Li, Jilin Oilfield General Hospital; Guozhong Li, The First Clinical College of Harbin Medical University; Yulan Zhu, The Second Clinical College of Harbin Medical University; Zichao Yang, The Fourth Clinical College of Harbin Medical University; Fengmin Yang, Daqing Oilfield General Hospital; Jun Zhou, Mudan Jiang Second hospital of Hailongjiang Province; Minxia Guo, Shaanxi Provincial People's Hospital; Zhengyi Li, The First Afflicated Hospital of Medical College of Xian Jiaotong University; Qilin Ma, The First Hospital of Xiamen; Renbin Huang, Chenzhou First People's Hospital; Bo Xiao, Xiangya Hospital of Centre-south University; Kangning Chen, Southwest Hospital; Xinyue Qin, The First Affiliated Hospital of Chongqing Medical University; Changlin Hu, The Second Affiliated Hospital of Chongqing Medical University; Li Gao, Chengdu Third Municipal People's Hospital; Jinsheng Zeng, The First Affiliated Hospital of Sun Yat-Sen University; Anding Xu, The First Affiliated Hospital of Jinan University; Xiong Zhang, Guangdong People's Hospital; Ming Shao, The First Affiliated Hospital of Guangzhou Medical University; Feng Qi, LiWan Hospital of GuangZhou Medical College; Weimin Xiao, Dungun Municipal People's Hospital; Suping Zhang, Guangzhou Red Cross Hospital; Xiaoping Pan, Guangzhou First TMUNICIPAL People's Hospital; Suyue Pan, Nan Fang Hospital; Yefeng Cai, Guangdong Provincial Hospital of Traditional Chinese Medicine; Qi Wan, Jiang Su People's Hospital; Yun Xu, Drum Tower Hospital Affiliated to Nanjing Medical University Upper First-class Hospital; KaiFu Ke, he Affiliated Hospital of Nantong University Upper First class Hospital; Yuenan Kong,Wuxi Second People's Hospital Upper First-class Hospital; Qing Di, Neurology Hospital Affiliated to Nanjing Medical University Upper First-class Hospital; Fengyang Shao, Jiangsu Province Lianyungang Hospital of TCM Upper First-class Hospital; Yajun Jiang, Jiangsu Province Hospital of TCM Upper First-class Hospital; Daming Wang, The First People's Hospital of Changzhou Upper First-class Hospital; Li Guo, The Second Hospital of Hebei Medical University; Wencui Xue, Qinhuangdao C.

Appendix B: Institutional review board within the CNSR network

Institutional review board at Beijing Tiantan Hospital; Institutional review board at Beijing Anzhen Hospital; Institutional review board at Beijing Military District Gengral hospital of Chinese People's Liberation Army; Institutional review board at Peking Union Medical College Hospital of Peking University; Institutional review board at Beijing Jishuitan Hospital; Institutional review board at Beijing Shijitan Hospital; Institutional review board at Peking University Third Hospital; Institutional review board at Beijing Xuanwu Hospital; Institutional review board at Beijing Friendship Hospital Affiliated to Capital Medical University; Institutional review board at Beijing Guangwai Hospital; Institutional review board at Beijing Shunyi District Hospital; Institutional review board at Beijing Tongren Hospital; Institutional review board at Shanghai Renji Hospital; Institutional review board at Shanghai First Municipal People's Branch hospital; Institutional review board at Zhongshan Hospital of Fudan University; Institutional review board at Xin Hua Hospital Affiliated to Shanghai Jiao Tong University; Institutional review board at the sixth People'sHospital Affiliated to Shanghai JiaoTong University; Institutional review board at Shanghai Ninth People's Hospital Affiliated to Shanghai JiaoTong University; Institutional review board at ShanghaiRuijin Hospital; Institutional review board at Shanghai 8th People's Hospital; Institutional review board at Pudong New Area People's Hospital; Institutional review board at Shanghai Yangpu Area Shidong Hospital; Institutional review board at Shanghai Yangpu Area Center Hospital; Institutional review board at Huashan Hospital of Fudan University; Institutional review board at Tianjin Medical University Gengeal Hospital; Institutional review board at Tianjin Huanhu Hospital; Institutional review board at Dagang Oilfield Gengeal Hospital; Institutional review board at Bohai Oilfield Hospital; Institutional review board at Baodi District People's Hospital of Tianjin; Institutional review board at The First Affiliated Hospital of China Medical University; Institutional review board at The First Afflicated Hospital of Dlian Medical University; Institutional review board at The Second Affiliated Hospital of Dlian Medical University; Institutional review board at Dlian Second People's hospital; Institutional review board at Dlian Third People's hospital; Institutional review board at Dalian Central hospital; Institutional review board at Dalian Economic and Technological Development District Hospital; Institutional review board at Shenying Military District General hospital of Chinese People's Liberation Army; Institutional review board at Shenyang Military District 202 Hospital; Institutional review board at National Traditional Chinese Medicine (TCM) Thrombus Treatment Center of Liaoning Province; Institutional review board at En'liang hopital of Tai'an County; Institutional review board at The First Clinical College of Jilin University; Institutional review board at Jilin Chemical Industrial Group General hopital; Institutional review board at Jilin Oilfield General Hospital; Institutional review board at The First Clinical College of Harbin Medical University; Institutional review board at The Second Clinical College of Harbin Medical University; Institutional review board at The Fourth Clinical College of Harbin Medical University; Institutional review board at Daqing Oilfield General Hospital; Institutional review board at Mudan Jiang Second hospital of Hailongjiang Province; Institutional review board at Shaanxi Provincial People's Hospital; Institutional review board at The First Afflicated Hospital of Medical College of Xian Jiaotong University; Institutional review board at The First Hospital of Xiamen; Institutional review board at Chenzhou First People's Hospital; Institutional review board at Xiangya Hospital of Centre-south University; Institutional review board at Southwest Hospital; Institutional review board at The First Affiliated Hospital of Chongqing Medical University; Institutional review board at The Second Affiliated Hospital of Chongqing Medical University; Institutional review board at Chengdu Third Municipal People's Hospital; Institutional review board at The First Affiliated Hospital of Sun Yat-Sen University; Institutional review board at The First Affiliated Hospital of Jinan University; Institutional review board at Guangdong People's Hospital; Institutional review board at The First Affiliated Hospital of Guangzhou Medical University; Institutional review board at LiWan Hospital of GuangZhou Medical College; Institutional review

board at Dungun Municipal People’s Hospital; Institutional review board at Guangzhou Red Cross Hospital; Institutional review board at Guangzhou First TMUNICIPAL People’s Hospital; Institutional review board at Nan Fang Hospital; Institutional review board at Guangdong Provincial Hospital of Traditional Chinese Medicine; Institutional review board at Jiang Su People’s Hospital; Institutional review board at Drum Tower Hospital Affiliated to Nanjing Medical University Upper First-class Hospital; Institutional review board at the Affiliated Hospital of Nantong University Upper First class Hospital; Institutional review board at Wuxi Second People’s Hospital Upper First-class Hospital; Institutional review board at Neurology Hospital Affiliated to Nanjing Medical University Upper First-class Hospital; Institutional review board at Jiangsu Province Lianyungang Hospital of TCM Upper First-class Hospital; Institutional review board at Jiangsu Province Hospital of TCM Upper First-class Hospital; Institutional review board at The First People’s Hospital of Changzhou Upper First-class Hospital; Institutional review board at The Second Hospital of Hebei Medical University; Institutional review board at Qinhuangdao C hospital.
